# Supplementary material for: Revisiting cysteine protease function in Trypanosoma cruzi: implications for parasite egress and differentiation
Source: Microbiol Spectr. 2026 Apr 30;14(6):e04132-25. doi: 10.1128/spectrum.04132-25 (PMC13228002; doi:10.1128/spectrum.04132-25)
Supplement: Supplemental material — Fig. S1 to S4. [file spectrum.04132-25-s0001.pdf]

Supplemental Material of **Revisiting Cysteine Protease Function in *Trypanosoma cruzi*: Implications for Parasite Egress and Differentiation.**

Sara De Grandis[1,2], Anne Niggli[1], Delia Bogenstätter[1], Gaelle Lentini[1]

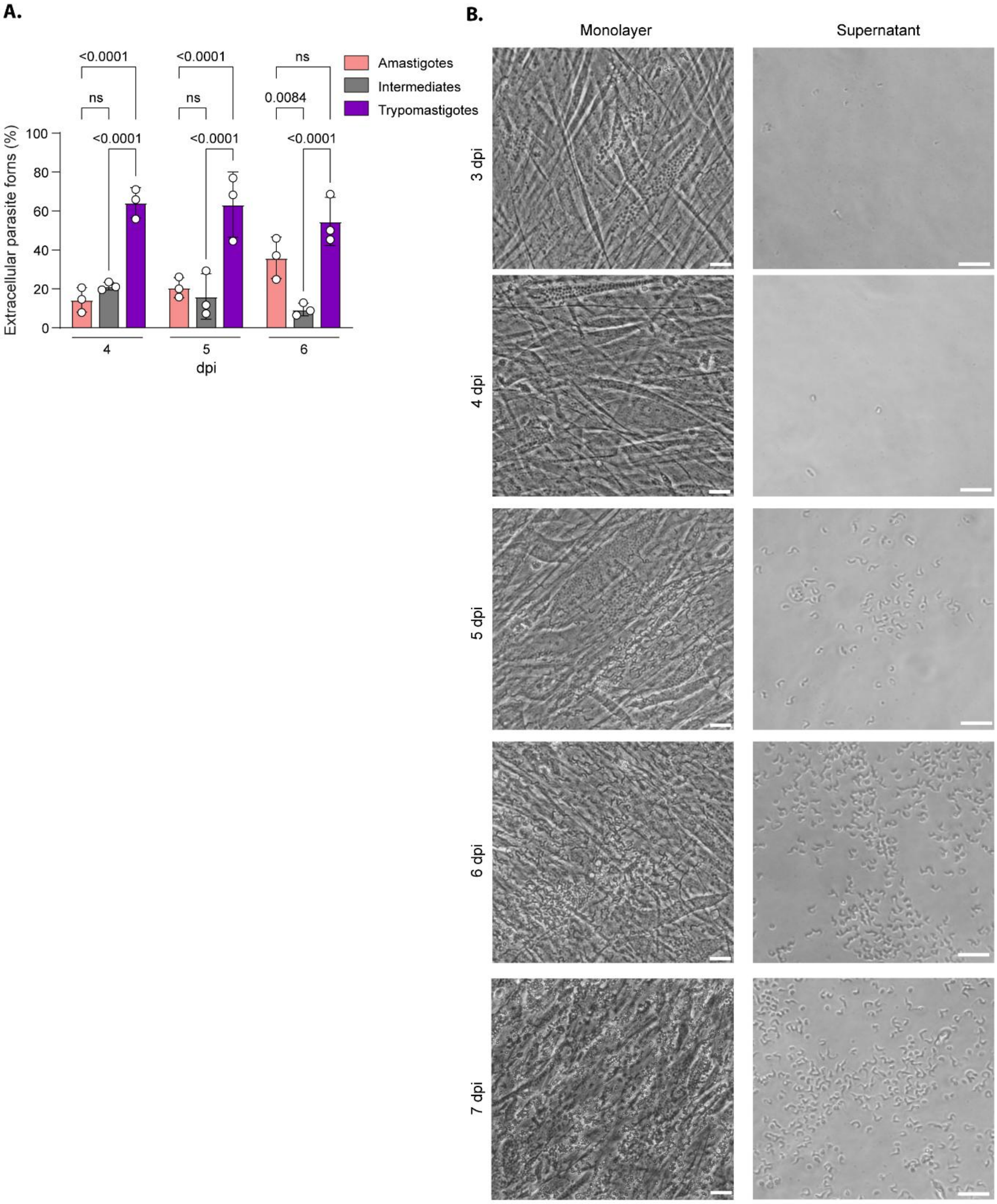

S1 Fig. Trypomastigotes represent the predominant form released from infected host cells. (A) Quantification of extracellular *T. cruzi* forms at 4, 5, 6 dpi. Statistical analysis was performed by two-way ANOVA with Tukey's multiple comparison. **(B)** Left panels: widefield images of infected HFF cultures at 3, 4, 5, 6, and 7 dpi. Right panels display the corresponding widefield images of culture supernatants. Scale bar= 20 μm.

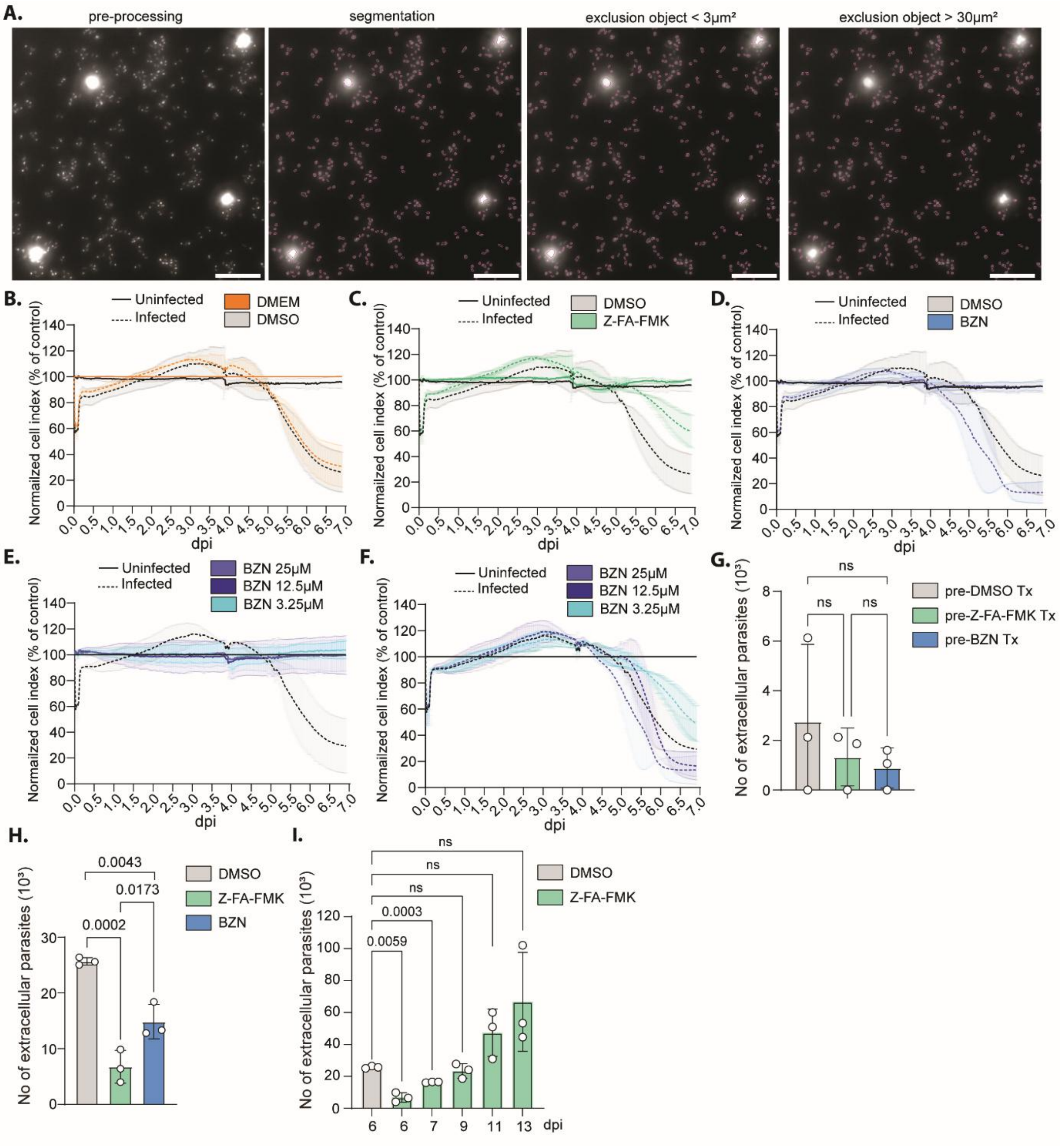

S2 Fig. Automated quantification of egress shows superior reproducibility compared to manual counting. (A) Representative widefield images of the supernatant from infected HFF cultures treated with DMSO at 6 dpi, stained with Hoechst, and acquired with the DAPI channel on the IN Cell Analyzer (Cytiva) pre- and post-processing. From left to right: raw image before processing in IN Cell Developer Software; segmented image showing the objects detected after applying a kernel size of 7 and sensitivity of 60; image showing the objects retained after applying a 3 μm^2 size exclusion filter; image showing

the objects retained after applying a 30 μm^2 size exclusion filter. Scale bar = 20um. **(B-D)** Kinetic measurement of cell index of uninfected and infected *T. cruzi* monolayer, treated or not with DMSO **(B)**, 50μM Z-FA-FMK **(C)** or 25μM BZN **(D)**, normalized to uninfected culture (mean ± SD, n = 4). **(E)** Kinetic measurement of cell index of uninfected culture treated with different concentration of BZN, compare to uninfected and infected *T. cruzi* monolayer (DMSO), normalized to uninfected culture (mean ± SD, n = 2). **(F)** Kinetic measurement of cell index of infected culture treated with different concentration of BZN, compare to uninfected and infected *T. cruzi* monolayer (DMSO), normalized to uninfected culture (mean ± SD, n = 2). **(G)** Graph showing the manual quantification of extracellular *T.cruzi* at 4 dpi in cultures prior to treatment (Tx). Statistical analysis was performed using ordinary one-way ANOVA followed by Tukey's multiple comparison (mean ± SD, n = 3). **(H)** Graph showing the manual quantification of extracellular *T.cruzi* at 6 dpi in cultures treated with DMSO, Z-FA-FMK or BZN. Statistical analysis was performed using ordinary one-way ANOVA followed by Tukey's multiple comparison (mean ± SD, n = 3). **(I)** Graph showing the manual quantification of extracellular *T. cruzi* at 6, 7, 9, 11, and 13 dpi in cultures treated with Z-FA-FMK compared to control condition (DMSO, 6 dpi). Statistical analysis was carried out using multiple unpaired t-tests (mean ± SD, n = 3).

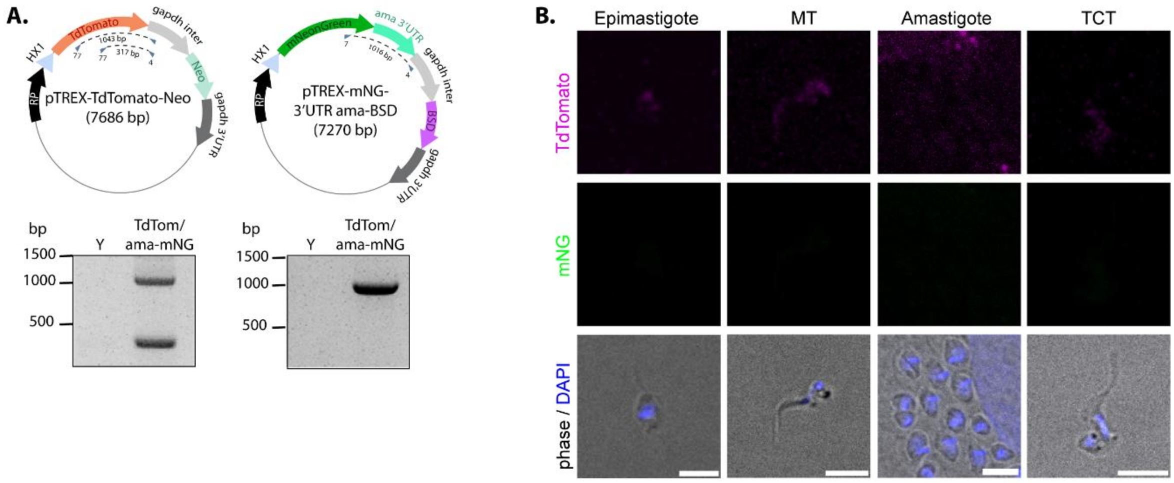

S3 Fig. Generation of TdTom/ama-mNG transgenic parasites, a dual-reporter strain. **(A)** Plasmid maps and PCRs showing the integration of the constructs TdTom-gapdh(3'UTR)-Neo and mNG-amastin(3'UTR)-BSD in the parasite genome. **(B)** Representative fluorescence images of the different *T. cruzi* life stages of the parental Y strain. Scale bar = 5µm.

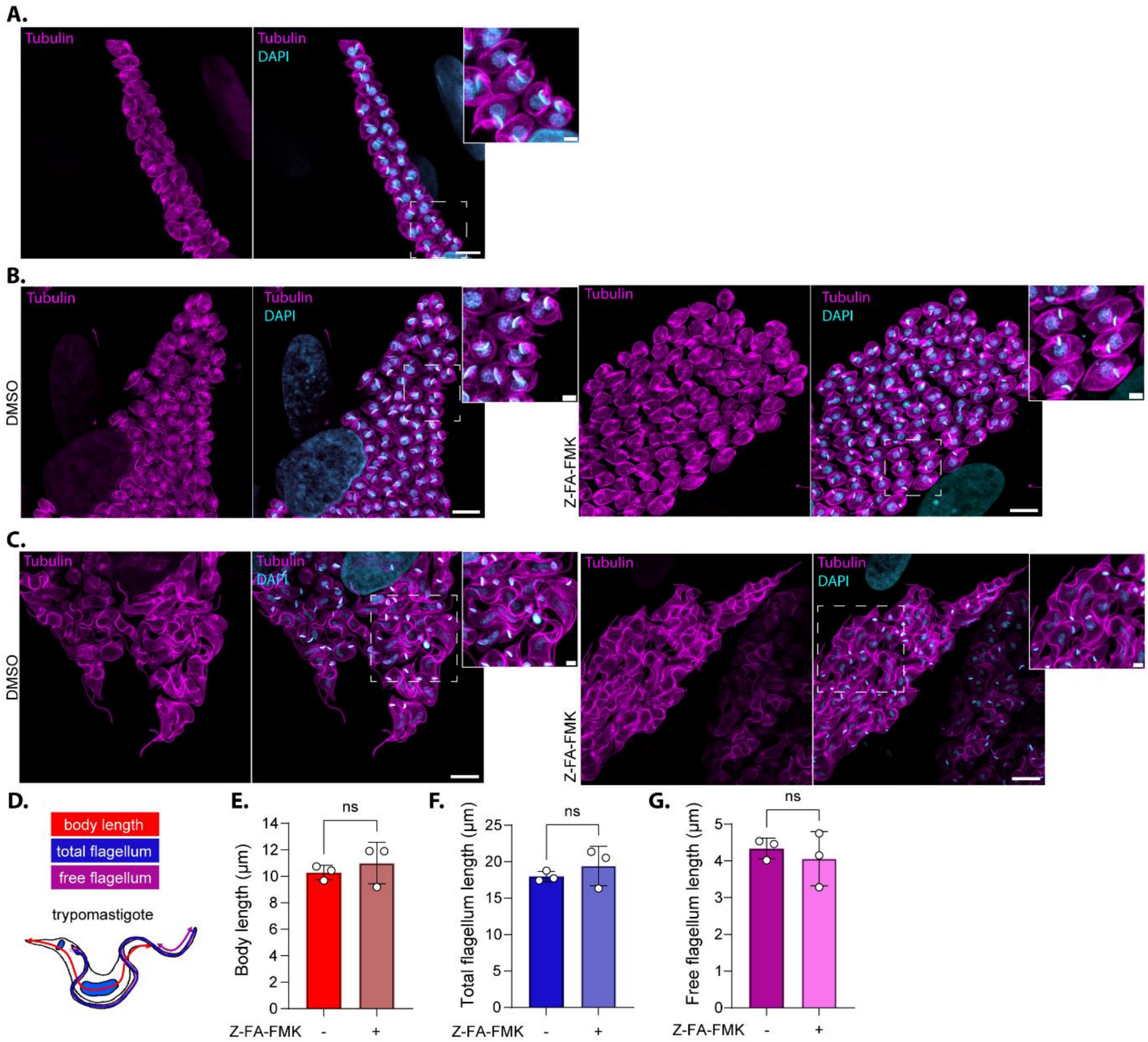

S4 Fig. Morphometric analyses performed on U-ExM samples. (A) Representative ultra-expansion microscopy images of infected cells at 4 dpi used for the morphometric analysis. DNA was stained with DAPI (cyan), and parasites are visualized using anti-tubulin antibodies (magenta). Scale bar = 20µm, Scale bar inset = 5µm. **(B)** Representative ultra-expansion microscopy images of infected cells containing amastigotes at 6 dpi, treated or not with Z-FA-FMK, used for the morphometric analysis. DNA was stained with DAPI (cyan), and parasites are visualized using anti-tubulin antibodies (magenta). Scale bar = 20µm, Scale bar inset = 5µm. **(C)** Representative ultra-expansion microscopy images of infected cells containing trypomastigotes at 6 dpi,

treated or not with Z-FA-FMK, used for the morphometric analysis. DNA was stained with DAPI (cyan), and parasites are visualized using anti-tubulin antibodies (magenta). Scale bar = 20μm, Scale bar inset = 5μm. **(D)** Schematic representation of the morphometric analysis performed on intracellular trypomastigotes from the U-ExM samples. **(E-G)** Graph representing the measurement of trypomastigote body length, total flagellum length and free flagellum length at 6dpi in culture treated or not with Z-FA-FMK (mean ± SD, n = 3). Statistical significance was assessed by unpaired t-test.
